# Clinical characteristics of pediatric tumor-induced osteomalacia: a systematic review and individual patient data analysis

**DOI:** 10.3389/fendo.2026.1871968

**Published:** 2026-07-21

**Authors:** Rong Chen, Zhangxin Wen, Cheng Cheng, Xinping Li, Jingyi Wang, Hong Liu, Hailing Chen

**Affiliations:** 1 Department of Osteoporosis, Beijing Jishuitan Hospital, Capital Medical University, Beijing, China; 2 Department of Metabolic Endocrinology, Zhuzhou Hospital Affiliated to Xiangya School of Medicine, Central South University, Zhuzhou, Hunan, China; 3 Laboratory for Clinical Medicine, Capital Medical University, Beijing, China

**Keywords:** hypophosphatemia, oncogenic ricket, pediatric tumor-induced osteomalacia, phosphaturic mesenchymal tumors, renal phosphate wasting

## Abstract

**Purpose:**

Pediatric tumor-induced osteomalacia (TIO), also called Tumor-induced rickets (TIR), is characterized by renal phosphate wasting, resulting in hypophosphatemia and rickets. This study aimed to evaluate the clinical characteristics of pediatric TIO patients.

**Methods:**

A systematic search was performed on December 26, 2025, in PubMed, Elsevier, SpringerLink, the China National Knowledge Infrastructure, and Wanfang Data using the terms: “tumor-induced rickets,” “tumor-induced hypophosphatemic rickets,” “oncogenic osteomalacia,” “hypophosphatemia,” “tumor-induced osteomalacia,” “children,” “child,” and “pediatrics”. Studies published in English and Chinese were included. This review was conducted according to the Preferred Reporting Items for Systematic Reviews and Meta-Analyses criteria.

**Results:**

A total of 62 pediatric TIO cases were included. Pediatric TIO was more frequent in males, who exhibited a higher proportion of weakness and deformities compared with females. 16.1% of patients were misdiagnosed with another condition. Somatostatin receptor-based imaging modalities demonstrated a high proportion for identifying these tumors. Pediatric TIO-causing neoplasms were predominantly located in the lower extremities and primarily originated from the bone tissue. Benign tumors were more common than malignant ones. The latter were associated with lower serum calcium levels. Surgery is the preferred definitive treatment for pediatric TIO, with an 84.2% resolution proportion.

**Conclusion:**

Pediatric TIO affects males more frequently than females, with male patients exhibiting a higher proportion of weakness and deformities, and is prone to misdiagnosis. Neoplasms are mostly located in the lower extremities and are predominantly benign. Surgery is the preferred treatment for pediatric TIO. For hypophosphatemic pediatric patients, TIO should be considered to preventing adverse outcomes.

## Introduction

Pediatric tumor-induced osteomalacia (TIO), also known as tumor-induced rickets (TIR), is a rare form of acquired hypophosphatemic rickets. It occurs in pediatric patients with open growth plates and is caused by abnormal secretion of fibroblast growth factor 23 (FGF23) by phosphaturic mesenchymal tumors (PMT) (1). Pediatric TIO is characterized by hypophosphatemia, impaired renal phosphate reabsorption, abnormal bone mineralization, with rickets serving as the predominant clinical manifestation (2). The tumors responsible for TIO are typically small and difficult to localize, often leading to diagnostic challenges and increased disease burden. Therefore, early identification, accurate localization, and timely intervention are crucial for improving patient prognosis. Owing to pediatric patients with TIO extreme rarity, research on pediatric TIO is mainly limited to case reports or reviews. Although previous studies have provided descriptive analyses of pediatric patients with TIO through case reports and literature reviews, these studies were small sample sizes (n = 14, n = 26) and lacked comparative analyses (3, 4).

Existing studies primarily focus on adult TIO. Pediatric TIO differs from its adult TIO in several aspects. In pediatric patients, persistent hypophosphatemia during skeletal growth impairs growth plate mineralization, resulting in skeletal deformities, growth retardation, and even short stature (2). If the diagnosis is delayed, these changes may be irreversible and affect the final height. Moreover, the differential diagnosis of hypophosphatemic rickets in pediatric is broader than in adults, including hereditary diseases that require long-term medical management rather than surgical intervention, such as X-linked hypophosphatemia (XLH) (3). Distinguishing pediatric TIO from these hereditary diseases in clinical presentation is challenging; however, the therapeutic implications are profound. Most tumors that cause TIO are benign and can be completely curable by surgical resection (5, 6). If patients with TIO are misdiagnosis as having hereditary disorders, they may undergo unnecessary lifelong medical therapy instead of tumor resection, thereby imposing substantial physical, psychological, and economic burdens on patients with TIO and their families (7, 8).

Consequently, this study aimed to conduct a systematic literature review and individual patient data (IPD) analysis to synthesize published global cases of pediatric TIO and investigate its epidemiological characteristics, clinical manifestations, diagnostic pathways, and therapeutic strategies. These findings may provide evidence-based insights into for the long-term care of pediatric TIO and enhance clinicians’ understanding and overall management of this rare disease.

## Materials and methods

### Study design and protocol

This systematic review was conducted and documented in accordance with the Preferred Reporting Items for Systematic Reviews and Meta-Analyses (PRISMA) guidelines. The review protocol was not registered.

This study was based exclusively on previously published literature and did not involve any human participants directly recruited by the authors.

### Eligibility criteria

The inclusion criteria were pediatric patients with TIO clinical diagnsis, who had open growth plates at disease onset; an onset age of < 18 years was also considered supportive but was not applied as a rigid cutoff. Studies were excluded if they involved primary hyperparathyroidism, Fanconi syndrome, or genetic disorders of phosphate homeostasis.

### Search strategy

The PICO question guiding the search was: What are the clinical characteristics of pediatric TIO? The PICO framework was defined as follows: P (population), pediatric patients with TIO; I (intervention), no intervention; C (comparison), no direct comparator; O (outcomes), clinical characteristics and outcomes. Subgroup comparisons were performed based on sex, tumor origin, and pathology.

The literature search was performed in several databases, including PubMed, Elsevier, Springer Link, the China National Knowledge Infrastructure, and Wanfang Data, up to December 26, 2025. The search used the terms: “tumor-induced rickets,” “Tumor-induced hypophosphatemic rickets,” “oncogenic osteomalacia,” “hypophosphatemia,” “tumor-induced osteomalacia,” “children,” “child,” and “pediatrics” (**Supplementary Document 1**). Only publications in English or Chinese were included. To identify additional relevant articles, the reference lists of all selected publications were manually reviewed.

### Study selection

**Figure 1:**
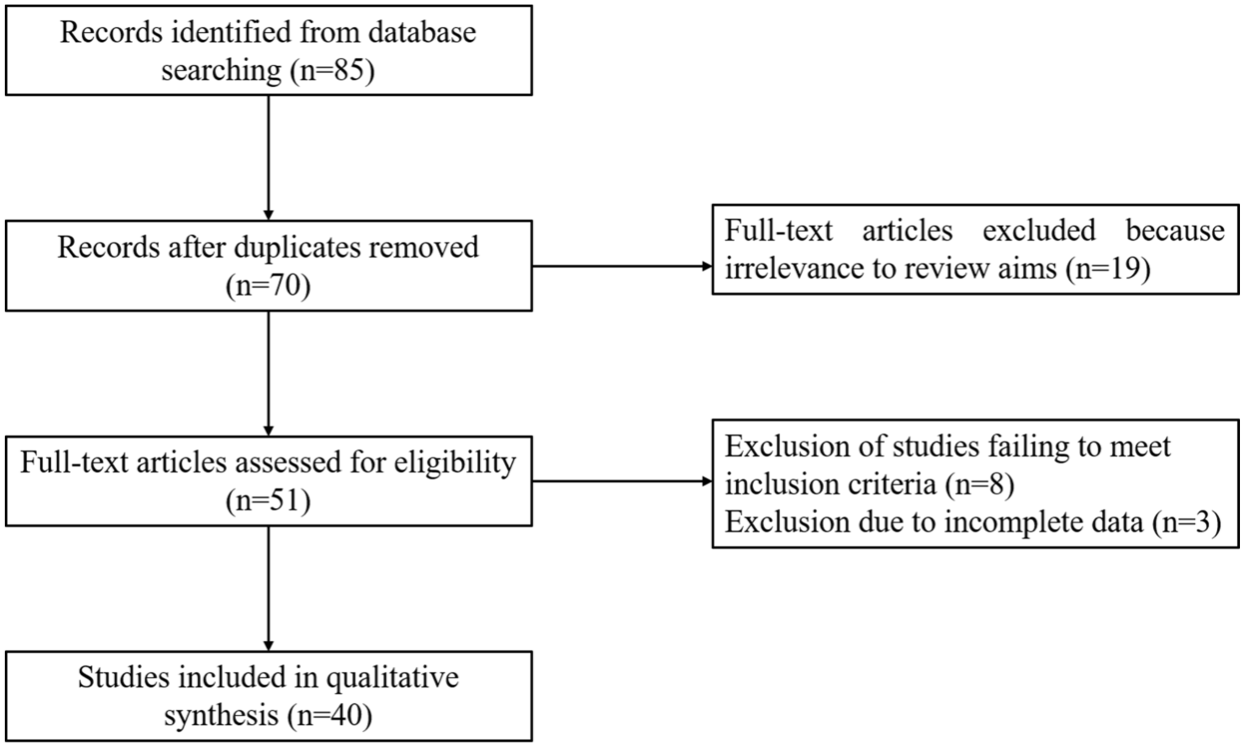
PRISMA flow chart of the study. PRISMA preffered reporting items for systematic reviews and meta-analyses.

This review included case reports, case series, and review articles. Review articles were selected to identify additional relevant studies through their reference lists that were not be found at the first research. Because earlier case reports might have been included as part of subsequent case series by the same authors, all articles were acquired and the references were analyzed to exclude duplicate data. Retrieved records were organized using EndNote (version X8) and Microsoft Excel (version 2024) for screening. Study screening and selection were performed by two independent reviewers (Rong Chen and Zhangxin Wen). Duplicate records were removed using EndNote and manually cross-checked. The initial screening based on titles and abstracts was performed by two independent reviewers. Subsequently, full texts of potentially eligible studies were retrieved and independently assessed against the criteria. Discrepancies between the two reviewers were resolved through discussion and consensus. If consensus could not be reached, a third senior reviewer (Hailing Chen) was consulted to make the final decision. The full process is documented in the PRISMA flowchart (**Figure 1**).

### Data extraction

The extracted data included authors, title, year of publication, age, sex, age at symptom onset, age at diagnosis, clinical manifestations, biochemical and imaging data, diagnostic tests, tumor characteristics, including location, histology, presence of metastasis, and location of metastasis, treatment type, and clinical outcomes. Data extraction was conducted by one author (Rong Chen) using Microsoft Excel and independently checked by a second author (Zhangxin Wen). Missing data were requested from the study authors. The risk of bias in the included studies was assessed using the Quality Assessment of Diagnostic Accuracy Studies-2 tool (9) (**Supplementary Document 2**).

### Definition of outcomes

In this study, treatment response was categorized as *remission*, *resolution*, *recurrence* and *persistence*. *Remission* was defined as partial improvement in clinical symptoms or radiographic signs after treatment, an increase in serum phosphate levels that did not reach the normal range after non-surgical treatment, or normalization of serum phosphate levels that remained dependent on pharmacological supplementation. *Resolution* was defined as the ultimate disappearance of symptoms, radiographic evidence of restored bone mineralization, and sustained normalization of serum phosphate levels without medical supplementation. *Recurrence* was defined as the relapse of hypophosphatemia after at least six months of normophosphatemia after surgical treatment. *Persistence* was defined as the persistent presence of hypophosphatemia and related symptoms after surgery or relapse within six months after tumor resection (10). Radiographic evidence of restored bone mineralization was defined as an improvement in skeletal abnormalities on X-ray (including healing of pseudofractures, improved trabecular and cortical bones, and alleviated growth plate abnormalities), and/or an increase in bone mineral density evaluated by dual-energy X-ray absorptiometry.

### Statistical analysis

The extracted data were compiled into a single database for analysis. Continuous variables are expressed as mean ± standard deviation or median (25^th^ and 75^th^ percentile). Categorical variables are presented as frequencies and percentages. Groups were compared using an independent two-sample t-test, a rank-sum test, and a chi-square or Fisher’s exact test for two independent samples.

All statistical analyses were conducted using Statistical Package for the Social Sciences software (version 25; IBM, Inc., Chicago, IL, USA). *P* ≤ 0.05 considered statistically significant.

## Results

Initially, a database search identified 85 records. After removing duplicates and screening the titles and abstracts, 70 full-text articles were assessed. Of these, 19 were excluded because they were irrelevant to the review aims. Of the remaining 51 articles assessed for eligibility, eight were excluded for not meeting the inclusion criteria, and five for incomplete data. Ultimately, 40 studies were included in the qualitative and quantitative syntheses, providing individual data for 62 participants (**Figure 1**, **Supplementary Document 3**).

### Baseline characteristics of pediatric patients

The baseline characteristics of 62 pediatric patients with TIO are presented in **Table 1**. Males accounted for most cases (37/62, 59.7%). The mean age at symptom onset was 9.8 years (range 0.75–18 years), and the mean age at diagnosis was 12.8 years (range 3–20 years). Bone pain (69.1%) and weakness (56.4%) were the most common symptoms. When stratified by sex, male pediatric patients with TIO exhibite a higher prevalence of weakness (67.6% versus 38.1%, *p* = 0.05) and a significantly higher proportion of skeletal deformities (58.1% versus. 19.0%, *p* = 0.009). Mean serum phosphorus was 0.60 ± 0.18 mmol/L and mean alkaline phosphatase (ALP) was 780.74 ± 602.36 U/L in female patients. For male patients, the mean serum phosphorus was 0.58 ± 0.15 mmol/L and mean ALP was 971.81 ± 613.42 U/L. Moreover, females exhibited vitamin deficiency with a mean 25-hydroxyvitamin D (25OHD) level of 16.69 ± 10.51 ng/mL, whereas males showed vitamin D insufficiency with an average 25OHD level of 23.09 ± 15.41 ng/mL. No significant sex-related differences were observed in biochemical parameters.

**Table 1:** Clinical and biochemical characteristics of pediatric TIO patients by sex.

Variable	n	Female (n=25)		Male (n=37)		*p*
		Results	Range	Results	Range	
Age at pediatric TIO onset, y	50	10.9 ± 4.0	2.8-18	9.2 ± 4.4	0.8-17.0	0.199
Age at pediatric TIO diagnosis, y	61	12.8 ± 5.5	3.0-23.0	12.8 ± 5.3	3.0-22.0	0.977
Duration of pediatric TIO, y	48	4.1 ± 5.0	0.1-19.0	4.2 ± 3.9	0.3-16.0	0.944
Clinical signs and symptoms						
**Weakness, n/%**	**55**	**8/38.1%**		**23/67.6%**		**0.050**
Bone pain, n/%	55	13/61.9%		25/73.5%		0.386
Functional impairment, n/%	55	8/36.4%		14/40.0%		1.000
Fracture, n/%	57	5/22.7%		11/31.4%		0.560
Pseudo-fracture, n/%	57	3/13.6%		5/14.3%		1.000
**Deformities, n/%**	**52**	**4/19.0%**		**18/58.1%**		**0.009**
Signs of rickets, n/%	49	13/76.5%		27/84.4%		0.700
Serum phosphate, mmol/L	56	0.60 ± 0.18	0.39-1.00	0.58 ± 0.15	0.29-1.13	0.612
Serum calcium, mmol/L	46	2.35 ± 0.13	2.05-2.52	2.37 ± 0.15	1.93-2.70	0.652
Serum ALP, U/L	50	780.74 ± 602.36	170.0-2191.0	971.81 ± 613.42	185.0-2400.0	0.287
25OHD, ng/mL	29	16.69 ± 10.51	10.3-27.0	23.09 ± 15.41	5.7-113.0	0.340
1,25(OH)_2_D_3_, pg/mL	25	14.0 (8.7, 18.9)	5.8-35.4	21.9 (9.5, 34.1)	4.3-55.0	0.396
FGF23, *ULN	20	2.20 (0.66, 3.49)	0.59-3.88	1.82 (0.77, 7.00)	0.47-651.59	0.458
TmP/GFR, mmol/L	31	0.44 ± 0.26	0.16-0.95	0.40 ± 0.13	0.15-0.65	0.677
PTH, pg/mL	42	93.5 ± 127.3	4.4-800.0	120.0 ± 167.9	6.0-900.0	0.598
Tumor size, cm	28	3.5 ± 3.5	1.2-11.5	5.1 ± 3.7	1.0-14.5	0.271
Cured, n/%	60	14/58.3%		26/72.2%		0.264

Bold values indicate statistically significant differences between groups.

TIO, tumor-induced osteomalacia; age at pediatric TIO onset, age at first presentation of pediatric TIO-related ricket syndrome; ALP, total alkaline phosphatase; 25OHD, 25-Hydroxyvitamin D; 1,25(OH)_2_D_3_, 1,25-Dihydroxyvitamin D_3_; FGF23, fibroblast growth factor 23; ULN, upper limit of normal; TmP/GFR, tubular maximum for phosphate corrected for glomerular filtration rate; PTH, Parathyroid Hormone.

Signs of rickets refers to radiographic or physical findings such as widened wrists/ankles, rachitic rosary.

### Diagnostic pathway

Initially, 10 (16.1%) patients were misdiagnosed with various conditions, including XLH, rheumatic diseases, myopathies, or osteoarticular disorders (**Supplementary Document 4**).

The causative tumor was successfully identified in 60 of the 62 patients, with a mean duration of 4.2 ± 4.2 years from symptom onset to tumor detection. Two patients who did not undergo localization examinations were clinically diagnosed, presenting clinical manifestations and supportive diagnostic findings consistent with hypophosphatemic rickets, and had no family history of rickets/osteomalacia. Moreover, clinical manifestations and supportive diagnostic findings improved after suspicious lesion resection, supporting the diagnosis of TIO. The two patients without identifiable tumors were excluded from the subsequent analysis.

### Tumor localization

Lesion detection was defined as the proportion of positive findings among all patients who underwent a specific examination. Functional imaging exhibited a high detection proportion for lesions. Positron emission tomography-computed tomography (PET-CT) with gallium-68 (^68^Ga)-1,4,7,10-tetraaza-cyclododecane-1,4,7,10-tetraacetic acid (DOTA)-somatostatin analogs localized all lesions in the eight patients who underwent this modality. In contrast, PET-CT with fluorine-18 (^18^F) fluorodeoxyglucose (FDG) identified tumors in seven of eight patients (87.5%). Octreoscan detected 12 of 15 lesions (80.0%), whereas bone scintigraphy detected three of five lesions (60.0%). Among anatomical imaging techniques, Magnetic resonance imaging (MRI) localized tumors in all 23 patients, computed tomography (CT) identified 20 of the 23 lesions (87.0%), and radiography detected 28 of 30 lesions (93.3%). Physical examination identified three out of three cases (100%), and ultrasound identified seven out of seven cases (100%).

### Tumor characteristics

**Figure 2:**
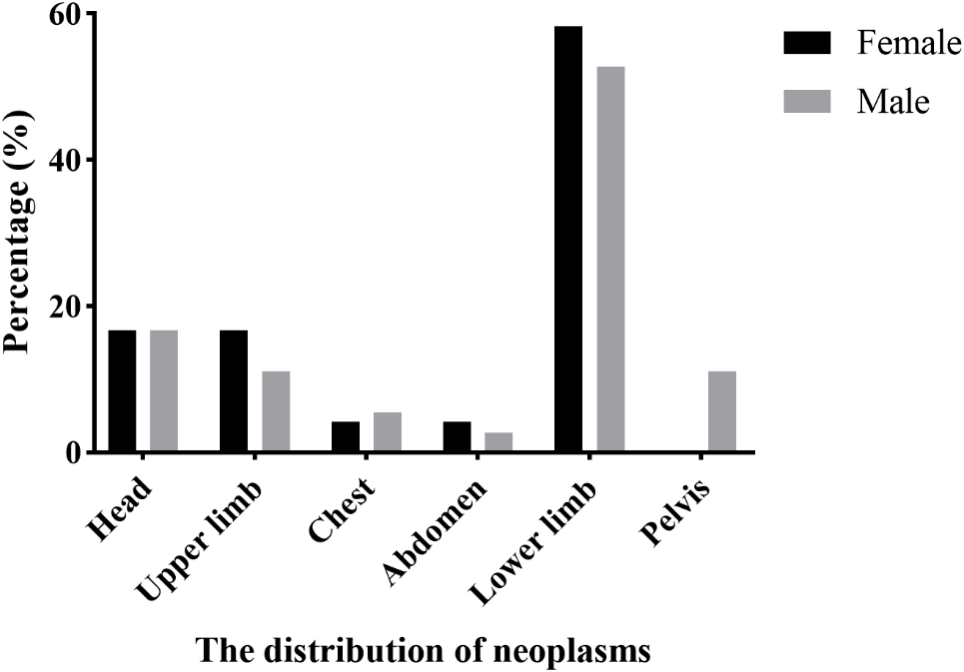
The distribution of neoplasm in pediatric tumor-induced osteomalacia by sex.

**Figure 2** summarizes the distribution of neoplasms in pediatric TIO. Overall, the lower limbs were the most frequently affected anatomical site (33 cases, 55%), followed by the head region (10 cases, 16.7%), with no significant differences between male and female patients (lower limb, 58.3% versus 52.8%; head, 16.7% versus 16.7%; both *p* > 0.05). Tumor distribution at other anatomical sites was similarly distributed between sexes.

**Table 2** details the clinical and biochemical characteristics of pediatric TIO according to tumor localization. Tumors originated from bone in 45 patients (75%) and from soft tissue in 15 patients (25%). Patients with soft tissue neoplasms exhibited a trend toward a higher prevalence of deformities (13/33.3% versus 7/63.6%, p=0.090) and lower serum phosphate levels (0.60±0.20mmol/L versus 0.54 ± 0.07mmol/L, p=0.083) than those with bone-localized tumors.

**Table 2:** Clinical and biochemical characteristics of pediatric TIO patients stratified by tumor tissue origin.

Variable	n	Bone (n=45)		Soft tissue (n=15)		*p*
		Results	Range	Results	Range	
Sex, F/M	60	20/25		4/11		0.224
Age at pediatric TIO onset, y	48	9.9 ± 4.0	2.8-18.0	9.9 ± 4.9	0.8-17.0	0.992
Age at pediatric TIO diagnosis, y	59	12.3 ± 5.0	3.0-22.0	13.9 ± 5.9	3.0-22.0	0.301
Duration of pediatric TIO, y	47	3.9 ± 4.2	0.2-19.0	4.2 ± 4.2	0.2-16.0	0.840
Clinical signs and symptoms						
Weakness, n/%	53	19/50%		11/73%		0.140
Bone pain, n/%	53	25/65.8%		11/73%		0.748
Functional impairment, n/%	55	15/36.6%		5/35.7%		1.000
Fracture, n/%	55	10/24.4%		5/35.7%		0.493
Pseudo-fracture, n/%	55	7/17.1%		1/7.1%		0.664
**Deformities, n/%**	**50**	**13/33.3%**		**7/63.6%**		**0.090**
Signs of rickets, n/%	47	29/85.3%		9/69.2%		0.237
**Serum phosphate, mmol/L**	**54**	**0.60** ± **0.18**	0.29-1.13	**0.54** ± **0.07**	0.42-0.68	**0.083**
Serum calcium, mmol/L	44	2.36 ± 0.15	1.93-2.70	2.36 ± 0.14	2.05-2.59	0.942
Serum ALP, U/L	49	933.5 ± 602.7	170.0-2400.0	894.3 ± 654.2	178.0-2268.0	0.846
25OHD, ng/ml	27	26.3 ± 22.4	7.1-113.0	17.5 ± 9.5	5.7-34.3	0.271
1,25(OH)_2_D_3_, pg/mL	25	14.9 (10.0, 33.0)	5.8-55.0	25.7 (10.4, 32.0)	7.7-43.3	0.674
FGF23, *ULN	20	3.69 (1.09, 7.00)	0.59-651.59	0.74 (0.61, 1.96)	0.47-3.08	0.296
TmP/GFR, mmol/L	29	0.43 ± 0.18	0.19-0.95	0.41 ± 0.17	0.15-0.65	0.858
PTH, pg/mL	40	115.0 ± 157.6	4.4-700.0	118.6 ± 156.8	6.0-523.0	0.950
Tumor size, cm	28	4.7 ± 3.9	1.2-10.5	4.2 ± 3.2	1.0-14.5	0.724
Cured, n/%	60	29/64.4%		11/73.3%		0.527

Bold values indicate a trend toward between-group statistical significance.

TIO, tumor-induced osteomalacia ; F, female; M, male; age at pediatric TIO onset, age at first presentation of pediatric TIO-related ricket syndrome; ALP, total alkaline phosphatase; 25OHD, 25-Hydroxyvitamin D; 1,25(OH)2D3, 1,25-Dihydroxyvitamin D_3_; FGF23, fibroblast growth factor 23; ULN, upper limit of normal; TmP/GFR, tubular maximum for phosphate corrected for glomerular filtration rate; PTH, Parathyroid Hormone.

Signs of rickets refers to radiographic or physical findings such as widened wrists/ankles, rachitic rosary.

Histological data were available from 60 subjects (**Supplementary Table 1**). Among these, seven cases (11.7%) were malignant, including osteosarcoma, renal cell carcinoma, aggressive osteoblastoma, malignant fibrosarcoma of soft tissue, and undifferentiated embryonal sarcoma of the liver. The remaining 53 cases (88.3%) were benign, of which 19 (31.7%) were PMTs and their mixed connective tissue variants (PMT-MCT). Patients with malignant pathology presented with significantly lower serum calcium levels (2.22 ± 0.19 mmol/L versus 2.38 ± 0.12 mmol/L, *p* = 0.006) and larger tumor lesions (8.5 (4.5, 13.0) cm versus 2.6 (1.8, 4.0) cm, *p* = 0.016) compared to benign cases (**Table 3**).

**Table 3:** Clinical and biochemical characteristics of pediatric TIO patients stratified by pathology.

Variable	n	Benign (n=53)		Malignant (n=7)		*P*
		Results	Range	Results	Range	
Sex, F/M	60	23/30		1/6		0.286
Age at pediatric TIO onset, y	48	9.8 ± 4.3	0.8-18.0	10.5 ± 4.14	6.0-16.0	0.719
Age at pediatric TIO diagnosis, y	59	12.6 ± 5.2	3.0-22.0	13.4 ± 5.4	7.0-22.0	0.720
Duration of pediatric TIO, y	46	3.4 ± 3.9	0.1-19.0	3.4 ± 5.7	0.3-16.0	1.000
Clinical signs and symptoms						
Weakness, n/%	**56**	28/57.1%		4/57.1%		1.000
Bone pain, n/%	56	35/71.4%		4/57.1%		0.742
Functional impairment, n/%	55	17/35.4%		3/42.9%		1.000
Fracture, n/%	55	14/29.2%		1/14.3%		0.710
Pseudo-fracture, n/%	55	7/14.6%		1/14.3%		1.000
Deformities, n/%	50	16/372%		4/57.1%		0.560
Signs of rickets, n/%	47	32/78.0%		6/100.0%		0.471
Tumor origin (Bone/Soft tissue)	60	41/12		4/3		0.486
Serum phosphate, mmol/L	54	0.55 (0.49, 0.61)	0.29-1.13	0.65 (0.55, 0.68)	0.42-0.81	0.113
**Serum calcium, mmol/L**	**44**	**2.38** ± **0.12**	2.14-2.70	**2.22** ± **0.19**	1.93-2.43	**0.006**
Serum ALP, U/L	49	935.1 ± 633.9	170.0-2400.0	837.7 ± 452.3	178.0-1350.0	0.719
FGF23, *ULN	20	1.67 (0.70, 4.96)	0.49-651.59	3.08 (3.08, 3.08)	3.08	0.795
TmP/GFR, mmol/L	29	0.39 (0.28,0.50)	0.15-0.95	0.42 (0.16, 0.55)	0.16-0.55	0.920
PTH, pg/mL	40	50.0 (23.5, 127.0)	4.4-700.0	230.0 (19.0, 252.0)	19.0-252.0	0.411
**Tumor size, cm**	**28**	**2.6 (1.8, 4.0)**	1.0-10.5	**8.5 (4.5, 13.0)**	3.5-14.5	**0.016**
Cured, n/%	60	38/71.7%		2/28.6%		0.065

Bold values indicate statistically significant differences between groups.

TIO, tumor-induced osteomalacia; F, female; M, male; age at pediatric TIO onset, age at first presentation of pediatric TIO-related ricket syndrome; ALP, total alkaline phosphatase; FGF23, fibroblast growth factor 23; ULN, upper limit of normal; TmP/GFR, tubular maximum for phosphate corrected for glomerular filtration rate; PTH, Parathyroid Hormone.

Signs of rickets refers to radiographic or physical findings such as widened wrists/ankles, rachitic rosary.

### Management of pediatric TIO

A total of 24 patients received medical therapies before tumor localization (**Supplementary Table 2**). Among them, 22 patients received phosphate combined with active vitamin D, including two patients who additionally received burosumab (an FGF23 antibody), one patient who received native vitamin D, two patients who received calcium supplements, and one patient who received other therapies. In this group, symptomatic or biochemical improvements were observed in 18 patients. Additionally, one patient received active vitamin D alone and achieved symptomatic and biochemical improvement. Another patient received calcium supplementation combined with native vitamin D, without in clinical and biochemical improvements.

**Figure 3:**
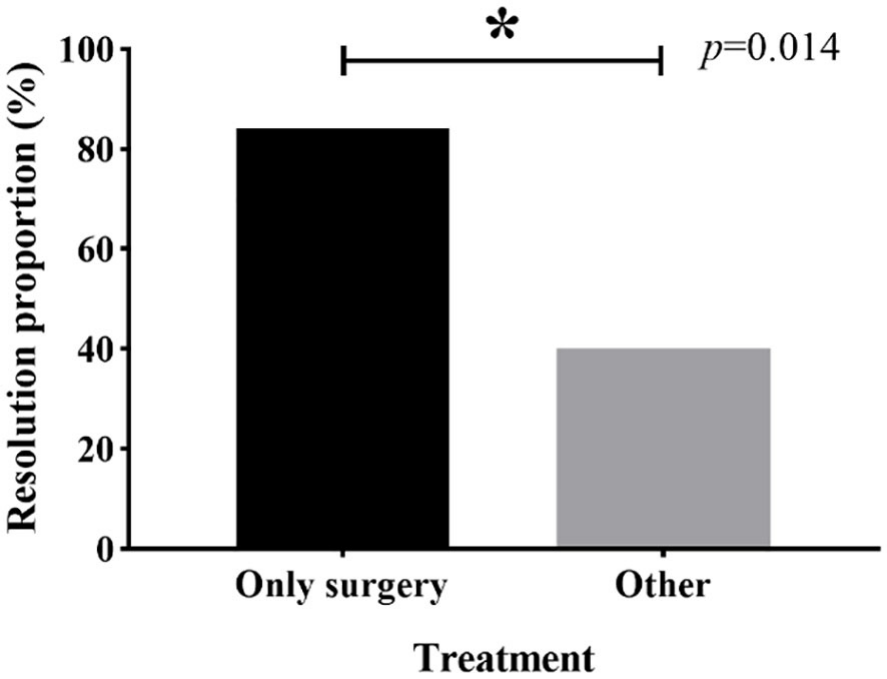
The resolution proportion of pediatric tumor-induced osteomalacia patients under different treatment modalities.

Management information was available for 48 pediatric patients with TIO. In the surgery-only group (n = 38), 32 patients (84.2%) achieved resolution, including two patients who achieved cure after reoperation following recurrence. Six patients (15.8%) achieved remission. No persistent disease or death was observed in this group. In contrast, among the 10 patients who received other treatment modalities, including non-surgical or combined treatment, four achieved resolution and five achieved remission or persistence, while one patient died due to pulmonary metastasis from malignant fibrosarcoma. No recurrence was observed in this group. The resolution proportion was significantly higher in the surgery-only group than in the other treatment groups (84.2% versus 40.0%, *p* = 0.014) (**Figure 3**).

## Discussion

This study provides a comprehensive characterization of pediatric TIO. Male patients presented a significantly higher proportion of weakness and skeletal deformities than female patients. Tumors predominantly arose in the lower extremities and originated from bone tissues, which findings may help guide clinical efforts in tumor localization. PMT and its mixed connective tissue variants were the major pathological types. Surgical intervention has a high cure proportion that suggest the importance of early and accurate tumor localization to reduce crippled rate and improve cure rate in this population.

The data indicated that pediatric TIO is more prevalent in males, who present with more pronounced clinical manifestations, including muscle weakness and skeletal deformities. This finding is consistent with the systematic review by Burchhardt et al. (3). The mechanism underlying these observations remains unclear and may be linked to sexual differentiation. Studies have reported that FGF23-secreting tumors may be more subtle in males, potentially causing longer disease duration and sustained hypophosphatemia (11, 12) and worsening skeletal and muscular damage.

In this study, 16.9% of patients with TIO were misdiagnosed with other diseases, including XLH and rheumatic diseases. Overlapping clinical, laboratory, and imaging features between pediatric TIO, XLH, and rheumatic disorders (13–15), may contribute to delayed diagnosis and increased long-term morbidity. Accordingly, serum phosphorus levels are recommended for individuals with musculoskeletal symptoms (16). An accurate distinction between TIO and XLH is essential for pediatric patients. Compared to XLH, pediatric patients with TIO have a relatively later onset and an absence of family history. Although both diseases can present with signs of rickets, patients with XLH are accompanied by dental abnormalities. Pediatric patients with TIO have more significant biochemical abnormalities than those with XLH. Xia et al. (17) reported that pediatric patients with TIO had lower serum phosphate, higher ALP and FGF23 levels than XLH patients, and suggested that pediatric TIO should be suspected when serum phosphate ≤ 0.59 mmol/L, ALP > 482 U/L, and FGF23 > 271.96 pg/mL. Furthermore, the two diseases have distinct diagnostic gold standards: TIO is diagnosed by pathology, such as PMT, whereas XLH is diagnosed by positive genetic testing for pathogenic genes including *PHEX* (**Supplementary Table 3**).

A stepwise tumor localization imaging, including functional and anatomical imaging, is recommended by guideline (6). Novel functional imaging techniques achieved a higher lesion detection proportion than traditional octreotide scans in this study, consistent with previous studies (18, 19). Its superior spatial resolution and high affinity for somatostatin receptors contribute to the identification of small or metabolically inactive lesions (18). Consequently, novel functional imaging modalities should be considered for pediatric patients with TIO who have highly suspicious but negative octreotide findings. Anatomical imaging can localize the tumor for precise surgical planning and should be chosen based on tumor location. Ultrasound or MRI is recommended for suspected soft tissue tumors, whereas X-ray or CT scans are recommended for bone lesions (20). FGF23 venous sampling is an additional method that provides both anatomical and functional hormonal information, with a localizing sensitivity of 94% (21). However, its invasive nature limits routine use, and it may be considered when advanced functional imaging is unavailable (21) when or multiple suspicious lesions are present to identify the responsible lesion (6). Multiple imaging screenings are required to identify causative tumors, making radiation safety a major concern in pediatric patients. Accordingly, accurate localization with fewer scans is essential for pediatric patients to reduce cumulative radiation exposure. Although functional imaging delivers a certain radiation dose, its diagnostic benefits outweigh potential radiation risks. To lower radiation burden without compromising detection, such scans use weight-based activity and low-dose CT for attenuation correction and anatomical localization (22). Radiation-free anatomical imaging, such as MRI and ultrasound, is preferred. If CT is unavoidable, the principle of as low as reasonably achievable should be strictly followed (23). X-rays are used for bone localization and must be accompanied by shielding. Comprehensive risk–benefit assessment can maximize diagnostic efficacy while minimizing long-term radiation hazards for pediatric TIO.

Tumors were predominantly located in the lower extremities in our cohort, accounting for over half of all lesions, followed by the head and neck region. This distribution is consistent with studies of pediatric and adult TIO cases (3, 4, 16, 24, 25), suggesting that tumor distribution may be similar across pediatric and adult populations. Accordingly, if the initial imaging fails to identify the causative lesion, attention should be directed to the lower extremities and the head or neck region.

In this cohort, most pediatric TIO-causing tumors originated from bone tissue, consistent with previous pediatric TIO reports (3, 4). This study also observed that patients with soft-tissue tumors exhibited a trend toward more skeletal deformities and a tendency toward lower serum phosphorus levels than those with bone-derived tumors. This is consistent with the findings of an adult TIO study (24) presenting that soft-tissue tumors exhibited a higher prevalence of deformities than bone tumors (17.4% versus 8.1%, *p* < 0.05). Although the precise mechanisms have not been fully elucidated, they are likely related to the inherent biological characteristics of the tumors. Soft-tissue tumors, particularly those located in deep areas, are frequently small and difficult to locate (26, 27), leading to delayed diagnosis. Ultimately, this can lead to severe clinical manifestations and biochemical disturbances.

Most tumors in this cohort were benign PMTs or PMT-MCT variants, aligning with previous studies (28, 29). This study revealed that pediatric patients with TIO and malignant tumors had lower serum calcium levels than those with benign lesions. This differs from studies in adult TIO cohorts, which reported that patients with malignant tumors had higher serum calcium levels (24, 28). We hypothesize that this discrepancy may be related to active skeletal growth in children. Rapid bone growth and severe mineralization defects impose a high calcium deposition demand on the developing skeleton (30). However, impaired intestinal calcium absorption reduces serum calcium levels (31). Additionally, malignant lesions in this study were larger than benign ones. The aggressive growth pattern of malignant tumors, including pronounced local invasiveness and the destruction of adjacent tissue (32), may also partially explain this discrepancy.

Surgical resection had a higher proportion of complete resolution than other interventions in our findings. A systematic review involving 1,493 patients with TIO reported that > 90% achieved biochemical remission following surgery (24). Complete surgical excision is the definitive and preferred therapy for TIO (16, 33), and wide-margin resection is crucial to prevent recurrence (16). Medical treatment is recommended for inoperable patients, those with recurrence or persistence, and those awaiting surgery (16). It alleviates symptoms, corrects biochemical abnormalities and prevents bone hunger syndrome. Conventional medical therapies, such as phosphate supplements and active vitamin D. The consensus on TIO recommends daily doses for pediatric patients: 20–40 mg/kg for phosphorus and 20–30 ng/kg for calcitriol (34). Biochemical parameters should be monitored every 3–6 months to guide dose adjustment. Treatment goals include elevating serum phosphorus, normalizing ALP, and maintaining PTH within the normal range. The novel therapy burosumab can effectively normalize phosphate metabolism, ameliorate bone deformity, and relieve symptoms in patients with TIO (35, 36). Hruska et al. (37) found that initial burosumab doses of 0.3–0.4 mg/kg every 2 weeks normalized serum phosphate levels in over 30% of pediatric patients with TIO, with a low risk of hyperphosphatemia (< 3%). Case reports supported that burosumab is effective and has a low incidence of adverse reactions in pediatric patients with TIO (38, 39). Moreover, Hidaka et al. (40) found that TIO patients aged 12–94 years treated with burosumab had lower ALP levels than those receiving active vitamin D and phosphate supplements therapy. Therefore, burosumab may be considered for patients who fail to achieve satisfactory responses to conventional medical therapies.

This study found that 25OHD levels were insufficient or deficient among pediatric TIO. Vitamin D deficiency/insufficiency can lead to secondary hyperparathyroidism and impair bone mineralization (41), potentially weakening treatment response to TIO therapy. Therefore, it is important to assess 25OHD levels at baseline and during follow-up, and to supplement vitamin D to maintain 25OHD within the sufficient range (typically ≥ 30 ng/mL or 75 nmol/L). However, when medical treatments are combined with native vitamin D supplements, serum and urinary calcium levels should be closely monitored for signs of hypercalcemia and hypercalciuria.

This study is the largest IPD cohort of pediatric TIO reported to date, but it has several limitations. First, the sample size is limited by the rarity of the disease. And the reported lesion detection proportion cannot serve as a sensitivity or specificity value. Given the insufficient follow-up data, long-term prospective studies are required to confirm our findings. Second, two patients lacked pathological confirmation for a definitive diagnosis; however, clinical manifestations and supportive diagnostic findings improved after suspicious lesion resection, supportive the diagnosis of TIO. Third, heterogeneity of included reports may limit data integration and affect the generalizability of the finding. The IPD approach relies on published case reports of varying qualities. Finally, the study included only Chinese and English literature, potentially limiting the comprehensiveness of results. Literature in other languages is recommended for future studies.

## Conclusion

Pediatric TIO affects males more frequently than females, with male patients exhibiting a high prevalence of muscle weakness and skeletal deformities. Pediatric TIO may be misdiagnosed due to non-specific symptoms, highlighting the importance of accurate differential diagnosis. The causative tumors were predominantly located in the lower extremities and commonly originated form bone tissue. Somatostatin receptor imaging has proven highly effective in tumor localization. Complete surgical resection is associated with an excellent prognosis. Therefore, although pediatric TIO is rare, it should be considered in patients presenting with rickets-like symptoms and acquired hypophosphatemic rickets beyond infancy to minimize misdiagnosis and diagnostic delay.

## Data Availability

All datasets generated during and/or analyzed during the current study are not publicly available but are available from the corresponding author on reasonable request. Requests to access these datasets should be directed to chenrong0918@163.com.
