# Supplementary material for: Clinical characteristics of pediatric tumor-induced osteomalacia: a systematic review and individual patient data analysis
Source: Front Endocrinol (Lausanne). 2026 Jul 21;17:1871968. doi: 10.3389/fendo.2026.1871968 (PMC13433172; doi:10.3389/fendo.2026.1871968)
Supplement: Supplementary file 1 [file DataSheet1.docx]

Supplementary Document 1. Search strategy.

**1、Search Strategy for PubMed**

("tumor-induced rickets"[MeSH Terms] OR "tumor-induced rickets"[Title/Abstract] OR "tumor-induced hypophosphatemic rickets"[Title/Abstract] OR "oncogenic osteomalacia"[Title/Abstract] OR "hypophosphatemia"[MeSH Terms] OR "hypophosphatemia"[Title/Abstract] OR "tumor-induced osteomalacia"[Title/Abstract]) AND ("child"[MeSH Terms] OR "child"[Title/Abstract] OR "children"[Title/Abstract] OR "pediatrics"[MeSH Terms] OR "pediatrics"[Title/Abstract])

Filters: Language: English, Chinese; Publication date: up to 2025.

**2、Search Strategy for Elsevier**

("tumor-induced rickets" OR "tumor-induced hypophosphatemic rickets" OR "oncogenic osteomalacia" OR "hypophosphatemia" OR "tumor-induced osteomalacia") AND ("child" OR "children" OR "pediatrics")

Filters: Language: English; Publication date: up to 2025.

**3、Search Strategy for Springer Link**

(tumor-induced rickets OR tumor-induced hypophosphatemic rickets OR oncogenic osteomalacia OR hypophosphatemia OR tumor-induced osteomalacia) AND (child OR children OR pediatrics)

Filters: Language: English; Publication end year 2025.

**4、Search Strategy for CNKI**

主题=((肿瘤诱导性佝偻病) OR (肿瘤诱导性低磷性佝偻病) OR (肿瘤性骨软化症) OR (低磷血症) OR (肿瘤诱导性骨软化症)) AND 主题=((儿童) OR (小儿) OR (儿科) OR (青少年))

**Filters**: Language: Chinese; Publication date up to 2025.

**5、Search Strategy for WanFang Data**

(关键词=肿瘤诱导性佝偻病 OR 关键词=肿瘤诱导性低磷性佝偻病 OR 关键词=肿瘤性骨软化症 OR 关键词=低磷血症 OR 关键词=肿瘤诱导性骨软化症) AND (关键词=儿童 OR 关键词=小儿 OR 关键词=儿科 OR 关键词=青少年)

Filters: Language: Chinese; Publication date up to 2025.
