# Supplementary material for: Clinical characteristics of pediatric tumor-induced osteomalacia: a systematic review and individual patient data analysis
Source: Front Endocrinol (Lausanne). 2026 Jul 21;17:1871968. doi: 10.3389/fendo.2026.1871968 (PMC13433172; doi:10.3389/fendo.2026.1871968)
Supplement: Supplementary file 2 [file DataSheet2.docx]

QUADAS-2

| Study |  | Risk of Bias | | | |  | Applicability concerns | | |
| --- | --- | --- | --- | --- | --- | --- | --- | --- | --- |
|  |  | Patient selection | Index test | Reference standard | Flow and timing |  | Patient selection | Index test | Reference standard |
| Morrell NT, et al.  (JBJS Case Connect. 2014) |  | L | L | L | H |  | L | L | L |
| Emodi O, et al  (Int J Oral Maxillofac Surg. 2018) |  | L | L | L | H |  | L | L | L |
| Carey DE, et al  (J Pediatr. 1986) |  | L | L | U | H |  | L | L | U |
| Ellis MB, et al.  (J Neurosurg Pediatr. 2016) |  | L | H | L | L |  | L | H | L |
| Aschinberg LC, et al.  (J Pediatr. 1977) |  | L | L | U | H |  | L | L | U |
| Ito N, et al.  (J Bone Miner Res. 2025) |  | L | H | L | L |  | L | H | L |
| Shulman DI, et al.  (J Pediatr. 2004) |  | L | U | L | L |  | L | U | L |
| Eyskens B, et al  (Eur J Pediatr. 1995) |  | L | L | H | H |  | L | L | H |
| Kumar S, et al.  (J Pediatr Endocrinol Metab. 2020) |  | L | L | L | H |  | L | L | L |
| Fernández-Cooke E, et al.  (Pediatrics. 2015) |  | L | L | H | H |  | L | L | H |
| Olivas-Mazón R, et al  (Pediatr Blood Cancer. 2020) |  | L | U | U | L |  | L | U | U |
| Crossen SS, et al.  (J Pediatr Hematol Oncol. 2017) |  | L | L | H | L |  | L | H | U |
| Farmakis SG, et al.  (Pediatr Radiol. 2015) |  | L | L | L | H |  | L | L | L |
| Xie Y, et al  (J Clin Endocrinol Metab. 2013) |  | L | L | U | L |  | L | L | U |
| Sakai Junior N, et al.  (Cancer Genet. 2012) |  | L | L | H | L |  | L | L | H |
| Amir G et al.  (J Pediatr. 1993) |  | L | L | H | L |  | L | L | L |
| Martini A, et al.  (Am J Dis Child. 1983) |  | L | L | L | H |  | L | L | L |
| Hanukoglu A, et al.  (Clin Pediatr (Phila). 1989) |  | L | L | U | L |  | L | L | L |
| Park YK, et al.  (J Korean Med Sci. 1994) |  | L | U | U | L |  | L | U | U |
| McCANCE RA, et al.  (Q J Med. 1947) |  | L | U | U | L |  | L | U | U |
| Parker MS, et al  (JAMA. 1981) |  | L | U | H | H |  | L | U | U |
| Sun JH, et al  (Chin J Practical Pediatrics. 2004) |  | L | U | U | L |  | L | L | L |

L: low risk. H: high risk. U: unclear risk.
