# Supplementary material for: Clinical characteristics of pediatric tumor-induced osteomalacia: a systematic review and individual patient data analysis
Source: Front Endocrinol (Lausanne). 2026 Jul 21;17:1871968. doi: 10.3389/fendo.2026.1871968 (PMC13433172; doi:10.3389/fendo.2026.1871968)
Supplement: Supplementary file 3 [file DataSheet3.docx]

Supplemetary Dcument 3. List of articles included in the systematic review with individual personal data analysis

S1. Lasnier-Siron J, Perret R, Jaafar A, Schaeverbeke T, Cetre NM. Oncogenic rickets diagnosed at age 8 and the risk of persistent rickets: a rare case of pediatric-onset tumor-induced osteomalacia. Osteoporos Int. 2025 Aug;36(8):1481-1485. doi: 10.1007/s00198-025-07469-2.
S2. Reyes-Múgica M, Arnsmeier SL, Backeljauw PF, Persing J, Ellis B, Carpenter TO. Phosphaturic mesenchymal tumor-induced rickets. Pediatr Dev Pathol. 2000 Jan-Feb;3(1):61-9. doi: 10.1007/s100240050008.
S3. Morrell NT, Beck NL, Clericuzio C, Frias-Kletecka C, Szalay EA. Tumor-Induced Rickets Presenting in an Adolescent: A Case Report and Review of the Literature. JBJS Case Connect. 2014 Sep 10;4(3):e79. doi: 10.2106/JBJS.CC.N.00006.
S4. Thi HN, Manh CP, Tuan LT, Le Thi LA, Thanh NN, Vilaiyuk S. Tumor-induced Osteomalacia in a Boy with Maxillary Ossifying Fibroma. J Clin Res Pediatr Endocrinol. 2023 Nov 22;15(4):421-425. doi: 10.4274/jcrpe.galenos.2022.2021-8-14.
S5. Emodi O, Rachmiel A, Tiosano D, Nagler RM. Maxillary tumour-induced osteomalacia. Int J Oral Maxillofac Surg. 2018 Oct;47(10):1295-1298. doi: 10.1016/j.ijom.2018.02.008.
S6. Carey DE, Drezner MK, Hamdan JA, Mange M, Ahmad MS, Mubarak S, Nyhan WL. Hypophosphatemic rickets/osteomalacia in linear sebaceous nevus syndrome: a variant of tumor-induced osteomalacia. J Pediatr. 1986 Dec;109(6):994-1000. doi: 10.1016/s0022-3476(86)80283-9.
S7. Ellis MB, Gridley D, Lal S, Nair GR, Feiz-Erfan I. Phosphaturic mesenchymal tumor of the brain without tumor-induced osteomalacia in an 8-year-old girl: case report. J Neurosurg Pediatr. 2016 May;17(5):573-7. doi: 10.3171/2015.9.PEDS14617. Epub 2016 Jan 1.
S8. Aschinberg LC, Solomon LM, Zeis PM, Justice P, Rosenthal IM. Vitamin D-resistant rickets associated with epidermal nevus syndrome: demonstration of a phosphaturic substance in the dermal lesions. J Pediatr. 1977 Jul;91(1):56-60. doi: 10.1016/s0022-3476(77)80444-7.
S9. Jiajue R, Ni X, Jin C, Huo L, Wu H, Liu Y, Jin J, Yu W, Lv W, Zhou L, Xia Y, Chi Y, Cui L, Pang Q, Li X, Jiang Y, Wang O, Li M, Xing X, Meng X, Xia W. Early Discrimination Between Tumor-Induced Rickets/Osteomalacia and X-Linked Hypophosphatemia in Chinese Children and Adolescents: A Retrospective Case-Control Study. J Bone Miner Res. 2021 Sep;36(9):1739-1748. doi: 10.1002/jbmr.4331.
S10. Burckhardt MA, Schifferli A, Krieg AH, Baumhoer D, Szinnai G, Rudin C. Tumor-associated FGF-23-induced hypophosphatemic rickets in children: a case report and review of the literature. Pediatr Nephrol. 2015 Jan;30(1):179-82. doi: 10.1007/s00467-014-2979-0.
S11. Ito N, Fukumoto S. Tumor-induced rickets/osteomalacia (TIO): diagnostic pitfalls and therapeutic options. J Bone Miner Res. 2025 May 24;40(5):572-576. doi: 10.1093/jbmr/zjaf047.
S12. Shulman DI, Hahn G, Benator R, Washington K, White KE, Farber J, Econs MJ. Tumor-induced rickets: usefulness of MR gradient echo recall imaging for tumor localization. J Pediatr. 2004 Mar;144(3):381-5. doi: 10.1016/j.jpeds.2003.11.023.
S13. Eyskens B, Proesmans W, Van Damme B, Lateur L, Bouillon R, Hoogmartens M. Tumour-induced rickets: a case report and review of the literature. Eur J Pediatr. 1995 Jun;154(6):462-8. doi: 10.1007/BF02029356.
S14. Kumar S, Shah R, Patil V, Ramteke-Jadhav S, Bal M, Lila A, Shah N, Bandgar T. Tumor-induced rickets-osteomalacia: an enigma. J Pediatr Endocrinol Metab. 2020 Jul 20:/j/jpem.ahead-of-print/jpem-2020-0079/jpem-2020-0079.xml. doi: 10.1515/jpem-2020-0079.
S15. Fernández-Cooke E, Cruz-Rojo J, Gallego C, Romance AI, Mosqueda-Peña R, Almaden Y, Sánchez del Pozo J. Tumor-induced rickets in a child with a central giant cell granuloma: a case report. Pediatrics. 2015 Jun;135(6):e1518-23. doi: 10.1542/peds.2014-2218.
S16. Xu M, Zhang Q, Peng D. A distal tibial tumor accompanied by tumor-induced osteomalacia. Orthop Surg. 2009 Aug;1(3):245-9. doi: 10.1111/j.1757-7861.2009.00035.x.
S17. Olivas-Mazón R, Martín-Cazaña M, Pérez-Mohand P, Garzón-Lorenzo L, Espino-Hernández M, Baro-Fernández M, Pérez-Alonso V. Tumor-induced osteomalacia in an adolescent with an undifferentiated embryonal sarcoma of the liver. Pediatr Blood Cancer. 2020 Jul;67(7):e28386. doi: 10.1002/pbc.28386.
S18. Crossen SS, Zambrano E, Newman B, Bernstein JA, Messner AH, Bachrach LK, Twist CJ. Tumor-induced Osteomalacia in a 3-Year-Old With Unresectable Central Giant Cell Lesions. J Pediatr Hematol Oncol. 2017 Jan;39(1):e21-e24. doi: 10.1097/MPH.0000000000000686.
S19. Farmakis SG, Siegel MJ. Phosphaturic mesenchymal tumor of the tibia with oncogenic osteomalacia in a teenager. Pediatr Radiol. 2015 Aug;45(9):1423-6. doi: 10.1007/s00247-015-3301-4.
S20. Xie Y, Li HZ. Oncogenic osteomalacia caused by renal cell carcinoma. J Clin Endocrinol Metab. 2013 Dec;98(12):4597-8. doi: 10.1210/jc.2013-3335.
S21. Asnes RS, Berdon WE, Bassett CA. Hypophosphatemic rickets in an adolescent cured by excision of a nonossifying fibroma. Clin Pediatr (Phila). 1981 Oct;20(10):646-8. doi: 10.1177/000992288102001005.
S22. Nuovo MA, Dorfman HD, Sun CC, Chalew SA. Tumor-induced osteomalacia and rickets. Am J Surg Pathol. 1989 Jul;13(7):588-99. doi: 10.1097/00000478-198907000-00007.
S23. Hasegawa T, Shimoda T, Yokoyama R, Beppu Y, Hirohashi S, Maeda S. Intracortical osteoblastic osteosarcoma with oncogenic rickets. Skeletal Radiol. 1999 Jan;28(1):41-5. doi: 10.1007/s002560050470.
S24. Ward LM, Rauch F, White KE, Filler G, Matzinger MA, Letts M, Travers R, Econs MJ, Glorieux FH. Resolution of severe, adolescent-onset hypophosphatemic rickets following resection of an FGF-23-producing tumour of the distal ulna. Bone. 2004 May;34(5):905-11. doi: 10.1016/j.bone.2003.12.025.
S25. Jung GH, Kim JD, Cho Y, Chung SH, Lee JH, Sohn KR. A 9-month-old phosphaturic mesenchymal tumor mimicking the intractable rickets. J Pediatr Orthop B. 2010 Jan;19(1):127-32. doi: 10.1097/BPB.0b013e32832f59cb.
S26. Sakai Junior N, Pereira MF, Kalil RK. A simple bone cyst of the distal humerus with a t(7;12)(q21;q24.3) in a patient with hypophosphatemic rickets. Cancer Genet. 2012 Oct;205(10):541-3. doi: 10.1016/j.cancergen.2012.08.002.
S27. Amir G, Boneh A, Tochner Z, Bar Ziv J. Widespread hemangiomatosis of bone associated with rickets: recovery after irradiation. J Pediatr. 1993 Aug;123(2):269-72. doi: 10.1016/s0022-3476(05)81701-9.
S28. Lee DY, Choi IH, Lee CK, Chung CY, Cho KH. Acquired vitamin D-resistant rickets caused by aggressive osteoblastoma in the pelvis: a case report with ten years' follow-up and review of the literature. J Pediatr Orthop. 1994 Nov-Dec;14(6):793-8. doi: 10.1097/01241398-199414060-00020.
S29. Martini A, Notarangelo LD, Barberis L, Galvanini G, Burgio FP, Lanzi G. Acquired vitamin D-resistant rickets caused by prolonged latency in appearance of bone tumor. Am J Dis Child. 1983 Dec;137(12):1205-6. doi: 10.1001/archpedi.1983.02140380065024.
S30. Hanukoglu A, Chalew SA, Sun CJ, Dorfman HD, Bright RW. Surgically curable hypophosphatemic rickets. Diagnosis and management. Clin Pediatr (Phila). 1989 Jul;28(7):321-5. doi: 10.1177/000992288902800705.
S31. Yoshikawa S, Nakamura T, Takagi M, Imamura T, Okano K, Sasaki S. Benign osteoblastoma as a cause of osteomalacia. A report of two cases. J Bone Joint Surg Br. 1977 Aug;59(3):279-86. doi: 10.1302/0301-620X.59B3.893505.
S32. Case records of the Massachusetts General Hospital. Weekly clinicopathological exercises. Case 29-2001. A 14-year-old boy with abnormal bones and a sacral mass. N Engl J Med. 2001 Sep 20;345(12):903-8. doi: 10.1056/NEJMcpc010029. Erratum in: N Engl J Med 2001 Dec 13;345(24):1784.
S33. Park YK, Unni KK, Beabout JW, Hodgson SF. Oncogenic osteomalacia: a clinicopathologic study of 17 bone lesions. J Korean Med Sci. 1994 Aug;9(4):289-98. doi: 10.3346/jkms.1994.9.4.289.
S34. Moncrieff MW, Brenton DP, Arthur LJ. Case of tumour rickets. Arch Dis Child. 1978 Sep;53(9):740-5. doi: 10.1136/adc.53.9.740.
S35. McCANCE RA. Osteomalacia with Looser's nodes (Milkman's syndrome) due to a raised resistance to vitamin D acquired about the age of 15 years. Q J Med. 1947 Jan;16(1):33-46.
S36. Pollack JA, Schiller AL, Crawford JD. Rickets and myopathy cured by removal of a nonossifying fibroma of bone. Pediatrics. 1973 Sep;52(3):364-71.
S37. Willhoite D. ACQUIRED RICKETS AND SOLITARY BONE TUMOR: THE QUESTION OF A CAUSAL RELATIONSHIP. Clin Orthop Relat Res. 1975 June 1;109:210-1.
S38. Parker MS, Klein I, Haussler MR, Mintz DH. Tumor-induced osteomalacia. Evidence of a surgically correctable alteration in vitamin D metabolism. JAMA. 1981 Feb 6;245(5):492-3. doi: 10.1001/jama.245.5.492.
S39. Qi Wt, Jiang Y, Xia Wb. Case report of tumor-induced rickets in a child. Chin J Osteoporos Bone Miner Res. 2025; 18(04):447-452.
S40. Sun JH, Zhang C, QU SQ, et al. A case report of tumor-induced rickets. Chin J Practical Pediatrics. 2004; (11):668.
