# Supplementary material for: Clinical characteristics of pediatric tumor-induced osteomalacia: a systematic review and individual patient data analysis
Source: Front Endocrinol (Lausanne). 2026 Jul 21;17:1871968. doi: 10.3389/fendo.2026.1871968 (PMC13433172; doi:10.3389/fendo.2026.1871968)
Supplement: Supplementary file 4 [file DataSheet5.docx]

**Supplementary Table 1**. The histological data of pediatric tumor-induced osteomalacia patients.

| **Malignant** | **n=7** |
| --- | --- |
| osteosarcoma | 3/5.0% |
| renal cell carcinoma | 1/1.7% |
| aggressive osteoblastoma | 1/1.7% |
| malignant fibrosarcoma of soft tissue | 1/1.7% |
| undifferentiated embryonal sarcoma of the liver | 1/1.7% |
| **Benign** | **n=53** |
| PMT/PMT-MCT | 19/31.7% |
| non-ossifying fibroma | 8/13.3% |
| ossifying fibromas | 2/3.3% |
| chondroblastoma | 1/1.7% |
| chondromyxoid fibroma | 1/1.7% |
| bone hemangioma | 1/1.7% |
| benign osteoblastoma | 2/3.3% |
| aneurysmal bone cyst | 1/1.7% |
| simple bone cyst | 1/1.7% |
| central giant cell lesions | 3/5.0% |
| benign fibro-osseous lesions | 6/10.0% |
| degenerative osteophyte | 1/1.7% |
| benign mesenchymal tumor | 1/1.7% |
| hemangiopericytoma | 3/5.0% |
| myositis ossificans | 1/1.7% |
| angiofibroma | 2/3.3% |

PMT, phosphate-urinating mesenchymal tumors; PMT-MCT, PMT-mixed connective tissue variant.

**Supplementary Table 2.** Clinical characteristics of patients treated with medication before lesions detection.

|  | Results | Range |
| --- | --- | --- |
| Sex, F/M | 8/16 |  |
| Age at pediatric TIO onset, y | 8.8±4.4 | 2.0-16.0 |
| Age at pediatric TIO diagnosis, y | 13.8±5.9 | 3.0-23.0 |
| Duration of pediatric TIO, y | 6.0±5.5 | 0.3-19.0 |
| Serum phosphate, mmol/L | 0.51±0.22 | 0.29-0.94 |
| Serum calcium, mmol/L | 2.37±0.14 | 2.05-2.61 |
| Serum ALP, U/L | 983.3±635.4 | 178.0-2400.0 |
| 25OHD, ng/mL | 26.9±26.0 | 7.1-113.0 |
| 1,25(OH)_2_D_3_, pg/mL | 12.7 (8.7, 27.0) | 4.3-55.0 |
| FGF23, *ULN | 3.69 (1.82,10.09) | 1.09-651.59 |
| TmP/GFR, mmol/L | 0.37±0.20 | 0.15-0.74 |
| PTH, pg/mL | 207.0±270.9 | 6.0-900.0 |
| Drug treatment plan and treatment response |  |  |
| Treatment based on phosphate supplementation and active vitamin D analogues | 22 |  |
| Number of cases with effective treatment | 18 |  |
| Only active vitamin D analogues | 1 |  |
| Number of cases with effective treatment | 1 |  |
| Calcium and native vitamin D | 1 |  |
| Number of cases with effective treatment | 0 |  |

TIO, tumor-induced osteomalacia. Treatment response was defined as symptomatic and/or biochemical improvement.

**Supplementary Table 3.** Differential diagnosis between pediatric TIO and pediatric XLH.

| **Feature** | **XLH** | **Pediatric TIO** |
| --- | --- | --- |
| Etiology | Genetic | Acquired |
| Family history of hypophosphatemic rickets/osteomalacia | Positive | Negative |
| The age of onset | The two diseases manifest prior to epiphyseal closure; however, the onset of pediatric TIO usually occurs later than that of XLH | |
| Clinical manifestations | Symptoms of rickets accompanied by dental abnormalities | Symptoms of rickets |
| Growth | Short stature is common | Normal growth prior to onset |
| Biochemical indicates | The two diseases are characterized by hypophosphatemia, elevated ALP, and elevated FGF23; however, these alterations are significantly more marked in TIO | |
| Tumor Localization | Negative | Positive |
| Genetic Testing | *PHEX* mutation | Negative |
| Treatment | Medication | Surgery |

TIO, tumor-induced osteomalacia; XLH, X-Linked Hypophosphatemia; ALP, alkaline phosphatase; FGF23, fibroblast growth factor 23.
